# The Obstetrical Journal

**Published:** 1873-05

**Authors:** 


THE *Obstetrical Journal,* of Great Britain and Ireland, including Midwifery and the Diseases of Women and Children. Edited by James H. Aveling, M.D., and Alfred Wiltshire, M.D., with an American Supplement, edited by William F. Jenks, M.D. Henry C. Lea: Philadelphia. Five dollars per annum.

The first number of this very valuable publication has been received. It cannot fail to secure the indorsement of the entire profession, and should be liberally patronized. It is supported by an able and long list of the first men of Great Britain and Ireland.

